# Differential Response of Oyster Shell Powder on Enzyme Profile and Nutritional Value of Oyster Mushroom *Pleurotus florida* PF05

**DOI:** 10.1155/2014/386265

**Published:** 2014-06-26

**Authors:** Ram Naraian, Om Prakash Narayan, Jatin Srivastava

**Affiliations:** ^1^Department of Biotechnology, Mushroom Training and Research Centre, Faculty of Science, Veer Bahadur Singh Purvanchal University, Jaunpur, Uttar Pradesh 222003, India; ^2^Department of Biochemistry, Faculty of Science, Veer Bahadur Singh Purvanchal University, Jaunpur, Uttar Pradesh 222003, India; ^3^Department of Applied Science, Faculty of Environmental Science, Himalayan Institute of Technology and Management, BKT, NH-24, Lucknow, Uttar Pradesh 227005, India

## Abstract

Oyster mushroom* Pleurotus florida *was cultivated on different combinations of wheat straw (WS) as basal substrate and oyster shell powder (OSP) supplement. The OSP supplementation considerably responded to different cultivation phases. The mycelium grew fast and showed rapid growth rate (8.91 mmd^−1^) in WS + OSP (97 + 3) combination while WS + OSP (92 + 8) showed maximum laccase (3.133 U/g) and Mn peroxidase (MnP) activities (0.091 U/g). The climax level of laccase (5.433 U/g) and MnP (0.097 U/g) was recorded during fruit body initiation in WS + OSP (97 + 3) and WS + OSP (98 + 2) combinations, respectively. The WS + OSP (97 + 3) combination represented the best condition for mushroom cultivation and produced the highest biological efficiency (147%). In addition, protein and lipid contents in fruit bodies were slightly improved in response to OSP. The carbohydrate was significantly increased by raising concentration of OSP. The highest values of protein, carbohydrate, and lipid noted were 31.3 *μ*g/g, 0.0639 (g/g), and 0.373 (g/g) correspondingly. Conclusively it was evident that lower concentrations of OSP acted positively and relatively to higher concentrations and improved nutritional content which may suitably be used to enhance both yield and nutritional values of mushroom.

## 1. Introduction

Oyster mushrooms comprise the group of edible fungi with important medicinal biotechnological and environmental applications [1]. Bioconversion of lignocellulosic residues through cultivation of* Pleurotus* spp. offers the best prospect to utilize renewable resources in the production of protein rich food that will sustain food security for peoples [2, 3]. The versatility and absolute ease of cultivation coupled with oyster mushrooms that are edible have led to a great demand in recent years; as a result, oyster mushrooms are now the second largest produced mushrooms in the world [4]. Besides taste and nutritional appeal they secrete a wide range of enzymes which can degrade all key categories of lignocellulosic polysaccharides and pollutants.

The deficient supply of proper nutrients with natural lignocellulosic substrates dynamically affects different phases of mushroom cultivation [5]. Nutrient composition is one of the factors limiting saprobiotic colonization of cultivated mushrooms and particularly the fruiting of* Pleurotus* spp. [2, 6]. Various additives are recommended as supplements to the substrate for enhancement of oyster mushroom yield [2, 7, 8]. Yield can be intermittently raised by optimization of cultural conditions such as combining different substrates or adding supplements which have shortened the crop period for* Pleurotus* spp. and also increases mushroom productivity [2, 8, 9]. Recently farmers have been searching for an alternative substrate that may be more readily available and more cost effective or that may provide higher yields and better mushroom quality [9]. The oyster shell is not only good calcium enriched natural products but also a freely available waste [10].

In mushroom cultivation insoluble calcium salts are added to increase pH and thereby reducing bacterial contamination and to increase aeration by aggregation improving the texture and porosity of the substrate [10, 11]. The application of oyster shell powder (OSP) increases organic matter, available P, amino acids, and exchangeable cation concentrations in basal substrate. This is an elevated quality conditioner that consists of 96% calcium carbonate and many micronutrients [12]. It provides a long-lasting steady release of nutrients, helps to regulate pH levels, improves nutrients uptake, and enhances substrate tilt.

In the present task an effort was made to evaluate the influence of the powdered oyster shells in order to maximize mycelial growth rate, yield of the fruit bodies, biological efficiency, nutrients, and enzyme profile of oyster mushroom* Pleurotus florida* PF05.

## 2. Materials and Methods

### 2.1. Mushroom Strain and Maintenance

The white-rot fungus* Pleurotus florida* PF05 was employed in the present study. The culture was obtained from the Department of Microbiology, Institute of Bioscience and Biotechnology (IBSBT), Chhatrapati Shahu Ji Maharaj University, Kanpur, India. Culture was maintained in slant containing potato dextrose agar (PDA) medium (boiled potato 200 g/L, dextrose 20 g/L, and agar 20 g/L).

### 2.2. Collection of Oyster Shells and Preparation of Powder

Oyster shells were collected from the Gomati river bank (Barabanki, Uttar Pradesh, India) during summer season. These were washed and mechanically broken into small pieces followed by the liquid nitrogen treatment and ground to fine powder (size > 1 mm). The powder was sterilized chemically with 2% (v/v) formalin before supplementation.

### 2.3. Supplementation Regime

To evaluate the influence of oyster shell powder (OSP), it was accurately weighed to compose different combinations of WS and OSP including WS + OSP (99 + 1), WS + OSP (98 + 2), WS + OSP (97 + 3), WS + OSP (95 + 5), WS + OSP (92 + 8), and WS + OSP (90 + 10). These combinations were thoroughly mixed together prior to spawning and tested for various parameters.

### 2.4. Cultivation of Mushroom

The cultivation of mushroom was performed in polythene bags of standard size (18^″^ × 22^″^) by spawning through layer wise manner. The spawned bags were kept in dark at 23–25°C till complete mycelial covering. After complete mycelial run, polythene bags were gently torn apart and removed. The irrigation was started by water sprinkling and continued regularly at least thrice a day. The room atmosphere was preferably maintained between 70 and 90% relative humidity.

### 2.5. Harvesting and Determination of Biological Efficiency (BE)

The fruit bodies of appropriate size before they become overmatured were harvested with the help of sharp knife separately for each set and weighed. The cumulative biological efficiency was calculated as %BE with respect to Kg fresh mushroom Kg^−1^ dry substrate used.

### 2.6. Growth Rate Test

The growth rate test [13] of mycelia with response to different concentrations of oyster shell powder was conducted by the lineal growth assay as described earlier [2]. For this different substrate combinations were separately packed in 200 mm long (6 mm diameter) glass tubes to a density of approximately 0.8 g cm^−3^. The substrate was then inoculated to one end of the tube using 5 mm diameter mycelia disk. After inoculation both ends of tubes were plugged with sterile cotton plug and incubated at 22 ± 1°C in dark for 7 days. The mycelial growth was measured in mm and growth rate of each set was calculated after 7 days of incubation.

### 2.7. Preparation of Enzyme Extract

Crude enzyme extract was obtained by crushing 50 g of fermented substrate with 50 mL of 0.1 M phosphate buffer (pH 6.8). Mycelia and solid substrate were removed by filtration followed by centrifugation at 10,000 g in a cooling centrifuge (Remi, India) for 5 min. The supernatant obtained was employed for enzyme assay.

### 2.8. Enzyme Assay

Manganese peroxidase (MnP) activity was determined by using 3 mM phenol sulfonephthalein as substrate [14]. The reaction mixture containing buffer enzyme preparation, 2 mM H_2_O_2_, and phenol red was incubated for 5 min at 20 ± 1°C and absorbance was monitored at 610 nm, while the laccase activity was assayed [15] with 1 mM guaiacol (Sigma) prepared in 50 mM sodium acetate buffer (pH 4.2). Assay was performed at 22 ± 1°C and absorbance was measured at 465 nm. Enzyme activities were expressed in units (U) as *μ*mol product formed min^−1^ and are reported as Ug^−1^ solid substrate.

### 2.9. Determination of Nutrient Content (Protein, Carbohydrate, and Lipid) in Fruit Bodies

Total protein content present in fruit bodies was analyzed by the standard method of Bradford [16]. The solution of Coomassie Brilliant Blue G-250 dye was employed and absorbance was measured at 595 nm. The protein concentration was finally determined using standard curve plotted for bovine serum albumin (BSA) and calculated for mg/g of the fruit body sample. Total carbohydrate content available in fruit bodies was determined using the phenol-sulfuric acid method of Dubois et al. [17]. Total 500 *μ*L sample was mixed with 500 *μ*L 5% phenol followed by the addition of 2 mL sulfuric acid to the mixture and incubated for 20 minutes at room temperature. The absorbance was measured at 470 nm using spectrophotometer, and the total sugar was calculated by glucose standard curve. However, total amount of lipid was analyzed by the standard method by Folch et al. [18]. To analyze total lipid, 5 gram of each mushroom sample was suspended in 50 mL mixture of chloroform : methanol (2 : 1) and mixed thoroughly. The preparation was kept as such to stand for 3 days. The solution was filtrated and followed to centrifugation at 1000 g for 10 min. The upper layer of methanol was removed using Pasteur pipette and crude lipid was collected. The solvent was evaporated by heating and total lipid content was assessed gravimetrically.

### 2.10. Statistical Analysis

Analysis of variance (ANOVA) was performed to determine differences in each treatment using SAS for Windows 8.0; multiple comparison *t*-test for least significant differences (LSD) was conducted within columns to compare all means with the control of their respective treatment (*P* < 0.05).

## 3. Results and Discussion

### 3.1. Influence of OSP on Lineal Growth Rate of Mycelia

As shown in Table 1, the fastest mycelial growth rate (8.91 mmd^−1^) was recorded in response to the combination of WS + OSP (97 + 3), which was followed by 7.82 mmd^−1^ in WS + OSP (98 + 2) combination. However, unsupplemented set presented its optimal 4.66 mmd^−1^ growth rate. The higher OSP concentrations were found against mycelial growth, while lower levels were supportive and considerably stimulated the growth rate (Table 1). Similar observations were also reflected in a study [8] using* Pleurotus ostreatus* grown on sun flower seed hulls supplemented with N-NH^4+^ and/or Mn(II). In general OSP maximally contains CaCO_3_; however, some other minerals, namely, Al_2_O_3_, MgO, Na_2_O_3_, P_2_O_5_, and SiO_2_ are also found as traces [12]. The stimulating nature of the OSP at low levels might be due to the presence of these available micronutrients, while addition of these at high levels was suppressive that might be cause of the mineral accumulation at high amounts. On the other hand enhanced supplementation of additives increased the temperature of substrate that can suppress or kill the mycelia [9, 19] and reduces growth rate. The slow growth rates and low cell mass production associated with starved cultures result in long growth times and low yields, thus making it impractical for commercial production of enzymes.

### 3.2. Influence of OSP on Enzyme Profile

The addition of OSP at lower levels led to an increase in the activity of both enzymes. The highest units of laccase (3.133 Ug^−1^) and Mn peroxidase (0.091 Ug^−1^) were produced in WS + OSP (92 + 8) combination followed by the 2.865 Ug^−1^ and 0.085 Ug^−1^ in WS + OSP (95 + 5) combination comparatively which is many folds higher than unsupplemented sets. The lowest laccase and Mn peroxidase activities were recorded in the set of WS + OSP (85 + 15) combination added with the highest (15% w/w) OSP (data not shown). However, further increase in OSP concentration stimulated both laccase and Mn peroxidase (0.027 Ug^−1^) activities but up to a limit of WS + OSP (92 + 8) combination and reduced at higher levels (Table 1). Thus it can be stated that combination of WS + OSP (92 + 8) is the best supplementation condition for enzyme production. The addition of OSP which is generally rich in calcium ions generates porosity of substrate [7, 20] and consequently rapid growth of mycelium with limited lower levels. According to Totten Jr. et al. [21], oyster shell protein analysis reveals presence of several amino acids mainly valine, alanine, glutamine, serine, proline, leucine, tyrosine, lysine, and so forth. These amino acids in OSP might work like nitrogen source and stimulated the yield of enzymes. However, higher levels of OSP may increase pH and unfavorable temperature due to high N level in substrate that probably results in slow growth and finally low yield of enzymes.

### 3.3. Enzyme Profile during Different Phases of Fruit Body Development

**Figure 1:**
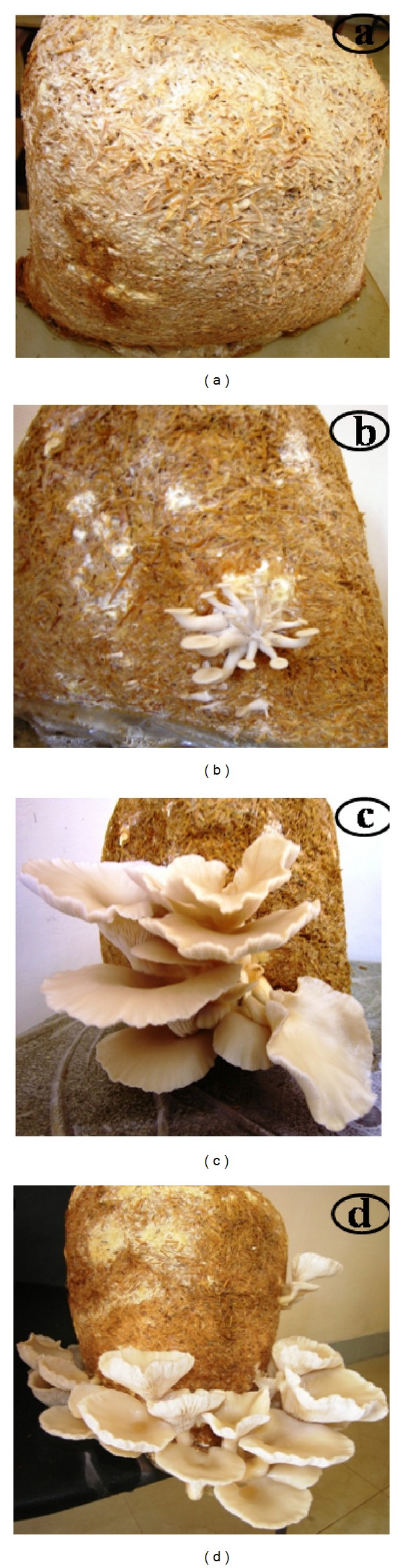
Different cultivation phases of oyster mushroom* Pleurotus florida* PF05, (a) complete mycelial run, (b) fruit body initiation, (c) flush-I, and (d) flush-II.

**Figure 2:**
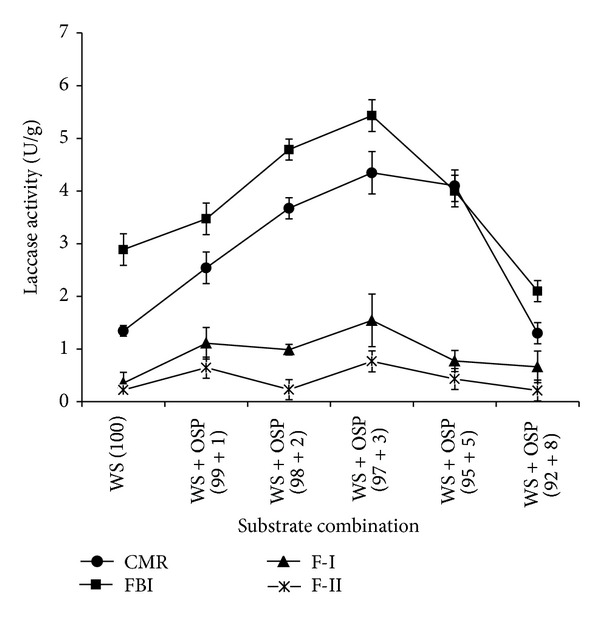
Influence of different OSP concentrations [0 (WS + OSP; 100 + 0), 1 (WS + OSP; 99 + 1), 3 (WS + OSP; 97 + 3), 5 (WS + OSP; 95 + 5), and 8 (WS + OSP; 92 + 8)%] on laccase profile of* Pleurotus florida* PF05 during different cultivation phases (CMR: complete mycelial run, FBI: fruit body initiation, F-I: flush-I, and F-II: flush-II).

**Figure 3:**
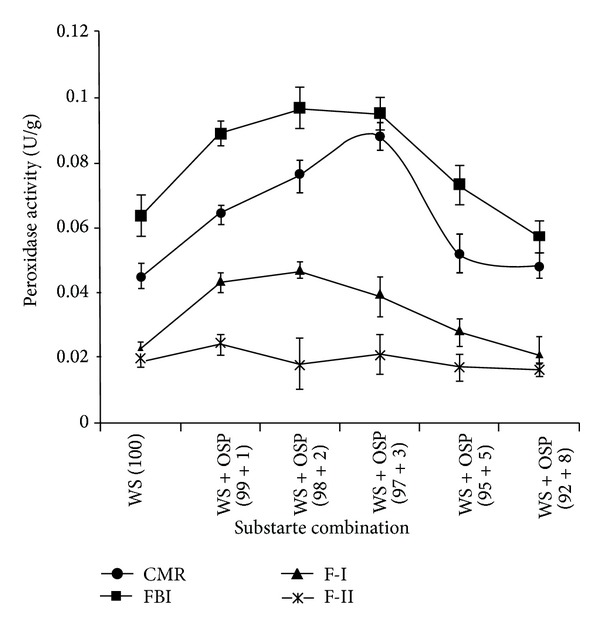
Influence of different OSP concentrations [0 (WS + OSP; 100 + 0), 1 (WS + OSP; 99 + 1), 3 (WS + OSP; 97 + 3), 5 (WS + OSP; 95 + 5), and 8 (WS + OSP; 92 + 8)%] on Mn peroxidase profile of* Pleurotus florida* PF05 during different cultivation phases (CMR: complete mycelial run, FBI: fruit body initiation, F-I: flush-I, and F-II: flush-II).

Different levels of OSP have extensively influenced the production of both enzymes during different phases of the mushroom cultivation. The expression of laccase and Mn peroxidase units were raised during fruit body initiation (FBI) in comparison to other phases. The highest units of laccase (5.433 Ug^−1^) were expressed in WS + OSP (97 + 3) combination and 0.097 Ug^−1^ units of Mn peroxidase in WS + OSP (98 + 2) combination. Similarly laccase transcription in* Lentinus edodes* peaked the highest during vegetative growth and declined at the fruiting stage [22]. Chen et al. [23] reported that laccase activity plays the most important role in the fruiting process. After fruit body initiation the enzyme activities were simultaneously reduced during harvesting (F-I and F-II) and the lowest units were expressed during the phase of flush-II of harvesting (Figures 1, 2, and 3). Xing et al. [5] also reported similar observations during cultivation of medicinal mushroom* Grifola frondosa*. Moreover, various other studies reported transcriptional regulation of laccase genes during developmental cycles of other mushrooms including* Lentinula edodes *and* Pleurotus abalonus* [24]. Peroxidase and laccase activities increased up to the formation of primordia, while they declined throughout the fruiting stage [25].

### 3.4. Influence of OSP on Biological Efficiency (BE)

**Figure 4:**
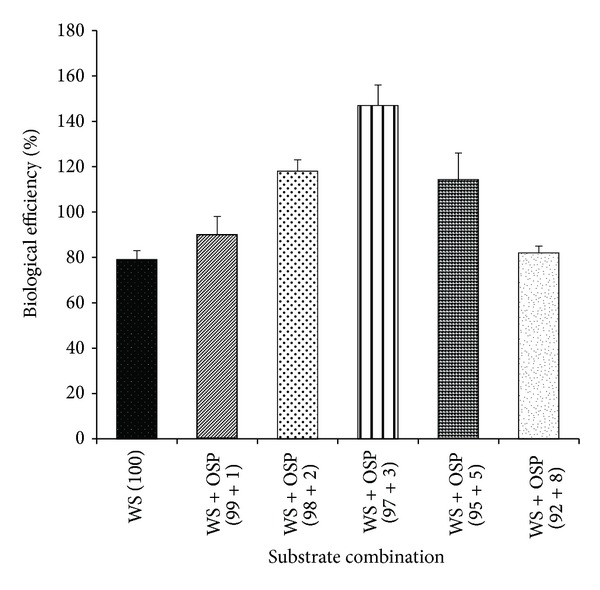
Influence of different OSP concentrations [0 (WS + OSP; 100 + 0), 1 (WS + OSP; 99 + 1), 3 (WS + OSP; 97 + 3), 5 (WS + OSP; 95 + 5), and 8 (WS + OSP; 92 + 8)%] on biological efficiency of* Pleurotus florida* PF05.

The biological efficiency of the mushroom was increased up to the level of WS + OSP (97 + 3) combination and reduced simultaneously at respective higher levels. The WS + OSP (97 + 3) combination was found to be the best substrate combination and resulted in the highest biological efficiency (147%, w/w) while the lowest biological efficiency (79%, w/w) resulted in unsupplemented set (Figure 4). It was also observed that lower levels of OSP were induced to other cultivation phases like complete mycelial run (CMR) and early primordial initiation (data not shown). Particularly similar observations regarding biological efficiency were also reported in corn cob and oil seed based substrates [2, 26]. The maximum amount of total yield was produced during the phase of first flush. However, fruit bodies obtained in the second and third flushes were comparatively lower to amounts of mushroom produced in first flush [9]. This might be due to diminishing nutrients available in the substrate [9].

### 3.5. Influence of OSP on Nutrient Content of Mushroom (Protein, Carbohydrate, and Lipid)

Influence of OSP on protein, carbohydrates, and lipid contents in matured fruit bodies was considerable. The different levels of OSP have considerably influenced nutrient contents including protein, carbohydrate, and lipid (Table 2). The nutrient contents studies were simultaneously increased with increasing the level of OSP, which was higher in contrast to unsupplemented set (WS + OSP; 100). However, higher level of OSP acted negatively and reduced nutritional contents (data not shown). The highest protein content (31.3 *μ*g/g) was noted in WS + OSP (92 + 8) combination. Similar level of protein content was also reported by other workers in* P. florida* and* P. ostreatus* mushrooms [2, 27]. Thus it can be speculated that availability of nutrients may affect the nutrient composition of fruit bodies [7]. Several kinds of amino acids are basically available in oyster shell [21] that may provide nutrients to fungus and might help in their protein synthesis.

## 4. Conclusions

Oyster shell, a by-product of shellfish waste, has a high potential to be used as a liming material in agriculture. Based on the findings of the present observation OSP can supply scarcity of nutrients for better growth and yield of mushroom. The lower levels of OSP were found supportive rather than higher for different cultivation phase, growth rate, enzyme profile, biological efficiency, and nutritive contents of fruit bodies. The supplementation of OSP at lower concentrations considerably improved the protein, lipid, and carbohydrate contents in fruit bodies. Conclusively lower levels of oyster shell powder could be used as an alternative limiting supplement to induce enzyme production and increase mushroom production.

## Figures and Tables

**Table 1:** Influence of different concentrations of OSP on mycelial growth rate and enzyme profile (laccase and Mn peroxidase) of *Pleurotus florida* PF05.

Substrate combinations	Growth rate (mmd^−1^)	Laccase activity (Ug^−1^)	Mn peroxidase (Ug^−1^)
WS (100)^c^	4.66 ± 0.04^c^	0.879 ± 0.006^c^	0.034 ± 0.001^c^
WS + OSP (99 + 1)	6.54 ± 0.07∗	1.467 ± 0.023∗	0.053 ± 0.004∗
WS + OSP (98 + 2)	7.82 ± 0.06∗	1.762 ± 0.031∗	0.061 ± 0.007∗
WS + OSP (97 + 3)	8.91 ± 0.12∗	2.227 ± 0.046∗	0.073 ± 0.009∗
WS + OSP (95 + 5)	7.55 ± 0.15∗	2.865 ± 0.048∗	0.085 ± 0.011∗
WS + OSP (92 + 8)	6.75 ± 0.08∗	3.133 ± 0.055∗	0.091 ± 0.009∗

WS: wheat straw.

OSP: oyster shell powder.

±: standard deviation (*n* = 8 for growth rate and *n* = 3 for enzymes).

∗Data are significantly different (*P* = 0.05) from their respective unsupplemented set in the same column.

^c^Controls.

**Table 2:** Influence of substrate and different concentrations of oyster shell powder on nutritional contents in fruit bodies of *Pleurotus florida *PF05.

Substrate combinations	Nutritive constituents in fruit bodies		
	Carbohydrate (g/g)	Lipid (g/g)	Protein (*µ*g/g)
WS (100)^c^	0.0411 ± 0.05^c^	0.0218 ± 0.02^c^	23.6 ± 0.3^c^
WS + OSP (99 + 1)	0.0455 ± 0.03	0.0273 ± 0.01∗	24.2 ± 0.5
WS + OSP (98 + 2)	0.0478 ± 0.06∗	0.0279 ± 0.05∗	24.7 ± 0.4∗
WS + OSP (97 + 3)	0.0529 ± 0.04∗	0.0311 ± 0.03∗	27.5 ± 0.3∗
WS + OSP (95 + 5)	0.0587 ± 0.07∗	0.0368 ± 0.06∗	31.3 ± 0.5∗
WS + OSP (92 + 8)	0.0639 ± 0.06∗	0.0373 ± 0.04∗	31.2 ± 0.7∗

±: standard deviation (*n* = 3).

∗Data are significantly different (*P* = 0.05) from their respective unsupplemented set in the same column.

^c^Controls.
